# Polymyalgia Rheumatica (PMR) with Normal Values of Both Erythrocyte Sedimentation Rate (ESR) and C-Reactive Protein (CRP) Concentration at the Time of Diagnosis in a Centenarian Man: A Case Report

**DOI:** 10.3390/diseases6040084

**Published:** 2018-09-20

**Authors:** Ciro Manzo

**Affiliations:** Internal and Geriatric Medicine Department, Azienda Sanitaria Locale Napoli 3 Sud, Rheumatologic Outpatient Clinic and Ambulatory of Geriatric Rheumatology, “Mariano Lauro” Hospital, 80065 Sant’Agnello, Italy; cirmanzo@libero.it; Tel.: +39-081-533-1465; Fax: +39-081-533-1447

**Keywords:** polymyalgia rheumatica, erythrocyte sedimentation rate, C-reactive protein, centenarians

## Abstract

The possibility that polymyalgia rheumatica (PMR) can be diagnosed when both ESR and CRP are normal at the time of diagnosis and before therapy with glucocorticoids, has been often discussed in the literature. We present a case report of a 100-year-old Caucasian man referred to our outpatient clinic, complaining of chronic pain in the shoulder and hip girdle associated with normal values of both ESR (21 mm/1st hour) and CRP (4 mg/dL). In the previous four months, several anti-inflammatory drugs and painkillers associated with physiotherapy treatments gave no significant improvement in pain and self-care. After an ultrasound (US) and an 18-fluorodeoxyglucose positron emission tomography associated with total body computed tomography (18-FDG PET/CT) examination, PMR was diagnosed and he started therapy with 17.5 mg prednisone, obtaining a fast improvement in pain and self-care. After 10 months of tapering, he stopped prednisone without relapse. During a 3-year follow-up, no alternative diagnosis was done. When a patient complains of chronic bilateral shoulder and hip girdle pain associated with normal inflammatory indices, it is reasonable to think in the first instance that this person is not suffering from PMR. Moreover, the possibility that PMR may onset in a centenarian person, is exceptional. In our patient, when we piece the puzzle together, the diagnosis of PMR was the most possible one.

## 1. Introduction

Polymyalgia Rheumatica (PMR) is a common inflammatory disease affecting older adults. Several investigators consider PMR as the most frequent inflammatory rheumatic disease in Caucasian persons older than 70-years. Its prevalence increases until the age of 90, with a slight decrease thereafter [1,2,3]. Its diagnosis is based upon recognition of a clinical syndrome consisting of pain and stiffness in the shoulder and pelvic girdle, associated with morning stiffness lasting at least 45 min. In some patients, systemic manifestations such as weight loss, fever of unknown origin, general malaise, loss of appetite, anemia are present [4]. An increase of erythrocyte sedimentation rate (ESR) at the time of diagnosis is present in all classification criteria [5,6], but it’s well known that in a proportion of PMR patients, from 7% up to 22%, ESR is normal. In these patients, C-reactive protein (CRP) values—when evaluated—are usually raised [7,8]. The possibility that in a patient with the aforementioned clinical manifestations, PMR is diagnosed, even if ESR and CRP are not increased, has still recently been questioned [9].

## 2. Case Report

**Figure 1:**
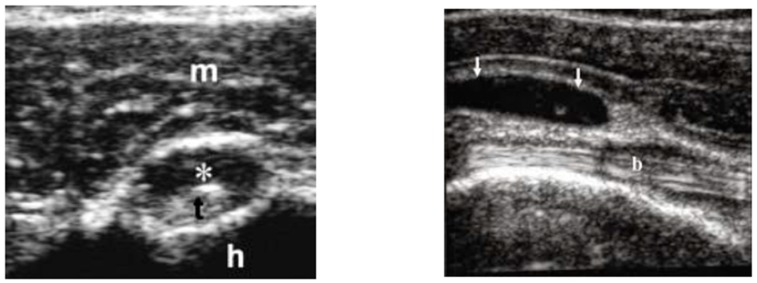
Long-head-biceps exudative tenosynovitis (left, *) and subdeltoid bursitis (right, arrows). m is for muscle; t is for long-head biceps tendon; h is for head (omeral head); b is for bursa.

**Figure 2:**
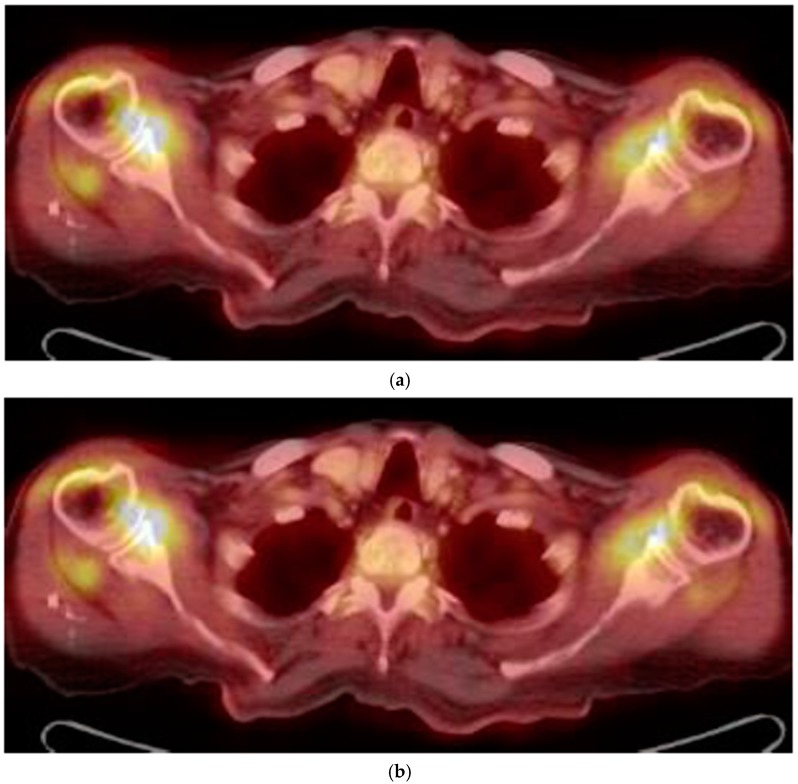
(**a**) FDG uptake in both the shoulder (18-FDG PET/CT fused axial slice); (**b**) FDG uptake in hips and subtrochanteric bursae.

In May 2015, a 100-year-old Caucasian man was referred to our outpatient clinic complaining of chronic pain in shoulder and hip girdle pain with 4-h morning stiffness. Constitutional manifestations were not present. In the previous four months, several non-steroid anti-inflammatory drugs (NSAIDs) and painkillers associated with physiotherapy treatments gave no significant improvement in pain and self-care. He had an X-ray of the chest, shoulders and pelvic, revealing no pathologic findings. An abdominal ultrasound (US) showed mild hepatomegaly and renal cysts. He did not suffer from psoriasis; no ocular, intestinal or uninary manifestations were present. ESR was 21 mm/1st hour (normal values < 30) at the onset and 12 mm/1st hour at the time of our examination. C-reactive protein (CRP) was 4 and 3 mg/dL (normal values < 6), respectively. Other laboratory data were all negative. In particular, serum fibrinogen levels were equal to 350 mg/dL (normal values < 400 mg/dL); rheumatoid factor (RF) and anti-protein citrullinated antibodies (APCA) were in their normal range; hemoglobin was equal to 12.8 gr/dL (normal values > 12.0 gr/dL); transaminases, creatine phospho kinase (CPK), protein electrophoresis, antinuclear cytoplasmic antibodies (ANCA) were in their normal ranges. Occult blood research in the stool was negative and fecal calprotectin dosage was in the normal range. Antibodies to hepatitis C virus and Australia antigen were absent. An US examination showed bilateral long-head-biceps exudative tenosynovitis and subdeltoid bursitis in his shoulders (Figure 1) and trochanteric bursitis in his right hip. An 18-fluorodeoxyglucose positron emission tomography (18-FDG PET) associated with total body computed tomography (CT) was performed (Figure 2) and excluded pathological findings in other sites. PMR was proposed. He started with 12.5 mg/day prednisone and there was a rapid improvement. After 10 days, he spontaneously stopped prednisone, but after 24 h, the manifestations reappeared, and he took it again. Prednisone tapering was made according to the schedule proposed by an international collaborative initiative [10] and stopped after 10 months. The patient refused a control PET/CT. A new US evaluation of shoulders and hips, performed after three months, showed normal findings. During a 3-year follow-up, he never had constitutional manifestations; no clinical manifestations suggestive for an overlapping giant cell arteritis (GCA) were observed; no alternative diagnosis was possible. As for today, our patient is fine.

## 3. Discussion

When a patient of greater than 50 years of age complains of chronic bilateral shoulder and hip girdle pain associated with normal inflammatory indices, it is reasonable to think in the first instance that this person is not suffering from PMR. In fact, there are several diseases with these clinical manifestations in which the values of inflammatory indices are normal at the time of diagnosis; among these, there are inflammatory (i.e., elderly onset rheumatoid arthritis), degenerative, infectious or neoplastic conditions [11]. For more, some of these can have a fast (but then transitory) response to systemic glucocorticoids (GCs) [11]. On the other hand, some PMR patients fail to achieve a complete response during therapy with GCs.

Additionally, some patients initially diagnosed with PMR may be reclassified as having a different disease during follow-ups, seronegative rheumatoid arthritis being the most common alternative [12]. Late-onset seronegative spondylarthropathy (LOSPA) is another PMR-mimicking disease (or vice versa) [13]. In our patient, the absence (at the time of diagnosis and during follow-up) of articular and/or extra-articular manifestations related to LOSPA associated with total ineffectiveness of NSAIDs were important elements to exclude it. Furthermore, in all guidelines for the treatment of seronegative spondyloarthropathy, low-dosed prednisone is never proposed as our patient used (with total remission of his clinical manifestations and no alternative diagnosis during a 3-year follow-up).

Up to now, in absence of a specific diagnostic test, the diagnosis of PMR remains clinical. In the last years, classification criteria proposed by a European League Against Rheumatism/American College of Rheumatology (EULAR/ACR) collaborative initiative highlighted US examination of both shoulders (showing glen-humeral synovitis, bursitis or biceps tenosynovitis) and hips (showing joint synovitis or trochanteric bursitis) [14]. 18-FDG PET/CT findings, even if not pathognomonic, are equally important for improving diagnostic accuracy [15,16]. In our patient, US examination and 18-FDG PET/TAC showed symmetrical inflammatory findings, exclusively located in his shoulder and hip girdles. CT findings of sacroiliac joints were reported as normal. Moreover, his pain and selfcare had a fast improvement after prednisone introduction with rapid relapse when he decided to suspend it. When we piece the puzzle together, the diagnosis of PMR was the only possible one. After a 3-year follow-up, no alternative diagnosis was found. What else could he have suffered from?

The reasons why ESR and CRP can be normal in an autoinflammatory disease such as PMR are only speculative. In PMR a non-specific inflammatory reaction is triggered by innate immunity activation [17]. Innate immunity may trigger fever, general malaise, fatigue and other constitutional manifestations. PMR patients with low ESR have a lower frequency of these compared to PMR with high ESR [18]. On the other hand, constitutional manifestations cause an increase in inflammatory indices [19]. In PMR patients, the absence of constitutional manifestations could be a result of interactions between innate and adaptive immunity within a specific genetic background [9,17,20].

According to a working hypothesis proposed by some investigators, those persons who are genetically predisposed to produce high levels of interleukin-6 (IL-6) during aging, have a reduced capacity to live long healthy lives. The genetic basis could be in a C/G polymorphism allocated at 5′-upstream of IL-6 (−174 C/G locus), with the GG genotype associated with higher levels of IL-6 serum levels and the CC or CG genotype associated with lower levels [21]. These differences could be the basis because serum CRP levels cannot increase in a centenarian man, when he has an auto-inflammatory disease such as PMR. In a longitudinal study including 684 Japanese centenarians, inflammation was the most important driver up to extreme old age [22]. Moreover, in a 100-year-old patient, ILs balance is different than in other age groups, with higher levels of anti-inflammatory cytokines such as IL-10 and TGF-beta [23].

According to our best knowledge, PMR in a 100-year-old is exceptional and studies regarding these suggested elements are very difficult.

Lastly, some investigators suggested that PMR with normal ESR and CRP could be an incomplete form of giant cell arteritis (GCA), manifested in the regions in the proximity of axillary, subclavian and/or femoral artery [24]. In GCA, not raised values of ESR and CRP are possible [25]. In our patient, this possibility was not proven.

## 4. Conclusions

Our case report draws attention to the existence of a non-frequent PMR with normal ESR and CRP at the time of diagnosis. In the clinical practice, this possibility should not stop the physician to include PMR in differential diagnosis. US and 18-FDG PET/CT evaluations might increase diagnostic rates of CRP- and ESR-negative PMR. A rigorous diagnostic work-up (with the exclusion of all confounding conditions) and an adequate follow-up are important [9,26].

## 5. Take-Home Messages

- The diagnosis of PMR is possible, even if ESR and CRP have not increased.
- US and 18-FDG PET/CT evaluations might increase diagnostic rates of ESR and CRP negative PMR.
- A rigorous diagnostic work-up and a long follow-up (one year, as minimum) are mandatory to avoid mistakes.
- The onset of PMR in a centenarian is exceptional. In this age range, the genetic background seems to be important in conditioning laboratory data more than in other periods of life.
